# High Diversity of *Sarcocystis* Species Identified in Intestines of Red Foxes (*Vulpes vulpes*) from Lithuania

**DOI:** 10.3390/ani16152285

**Published:** 2026-07-23

**Authors:** Tamara Kalashnikova, Naglis Gudiškis, Evelina Juozaitytė-Ngugu, Petras Prakas, Dalius Butkauskas, Donatas Šneideris

**Affiliations:** State Scientific Research Institute Nature Research Centre, Akademijos Str. 2, 08412 Vilnius, Lithuania; naglis.gudiskis@gamtc.lt (N.G.); evelina.ngugu@gamtc.lt (E.J.-N.); petras.prakas@gamtc.lt (P.P.); dalius.butkauskas@gamtc.lt (D.B.)

**Keywords:** *Sarcocystis*, red fox, *cox1*, *ITS1*, phylogeny, definitive host

## Abstract

*Sarcocystis* spp. are apicomplexan parasites with an obligatory two-host prey–predator life cycle. While the intermediate hosts (IHs) of many species are well known, the definitive hosts (DH) of numerous *Sarcocystis* spp., especially those involving wildlife predators, remain poorly understood. In the current study, *Sarcocystis* species richness was investigated in the intestines of 69 red foxes (*Vulpes vulpes*) from Lithuania. Intestinal samples were examined by light microscopy and nested PCR targeting the cytochrome c oxidase I gene (*cox1*) sequence of species associated with ungulate IH and the internal transcribed spacer I (*ITS1*) region of species using birds as IH, followed by Sanger sequencing and phylogenetic analyses. Red foxes harboured a broad spectrum of *Sarcocystis* species, with those utilising ungulate IHs being considerably more common than species associated with birds. Seven species, *Sarcocystis alces*, *Sarcocystis bertrami*, *Sarcocystis capreolicanis*, *Sarcocystis hjorti*, *Sarcocystis iberica*, *Sarcocystis linearis*, and *Sarcocystis morae*, were identified in red foxes for the first time. Nine previously reported species, *Sarcocystis albifronsi*, *Sarcocystis arieticanis*, *Sarcocystis capracanis*, *Sarcocystis cruzi*, *Sarcocystis gracilis*, *Sarcocystis miescheriana*, *Sarcocystis pilosa*, *Sarcocystis rileyi*, and *Sarcocystis tenella*, were also detected. In addition, two potentially novel *Sarcocystis* taxa were identified and warrant further investigation. These findings highlight the role of red foxes as an important DH and provide a valuable framework for comparative studies of *Sarcocystis* distribution in predatory mammals.

## Simple Summary

*Sarcocystis* parasites are widely distributed apicomplexan parasites, infecting reptiles, birds, and mammals. They are characterised by complex prey–predator life cycles, involving intermediate hosts, in which tissue sarcocysts develop, and definitive hosts, in which sexual reproduction and sporulation of oocysts occur. Pathogenicity of *Sarcocystis* is exposed in intermediate hosts, while definitive hosts are important for the transmission of these parasites. The red fox (*Vulpes vulpes*) is one of the most widespread and opportunistic predators in Europe and may play an important role in the maintenance and spread of these parasites. In this study, we investigated the prevalence and richness of *Sarcocystis* species in red foxes from Lithuania using microscopy and molecular methods. We identified a broad range of *Sarcocystis* species, including seven species recorded in red foxes for the first time and two potentially new taxa requiring further investigation. Parasites associated with ungulate intermediate hosts were considerably more common than those associated with birds, reflecting the feeding ecology of red foxes. Our findings demonstrate that the red fox is an important definitive host contributing to the circulation of *Sarcocystis* parasites in natural ecosystems.

## 1. Introduction

Members of the genus *Sarcocystis* are widely distributed apicomplexan parasites. There are more than 220 *Sarcocystis* species that have been described worldwide, infecting mammals, birds, and reptiles. They exhibit a dixenous life cycle, typically involving a prey–predator relationship. Asexual reproduction occurs in the tissues of the intermediate host (IH), leading to the formation of sarcocysts in muscles and other organs, including the CNS [1]. In the intestines of the definitive host (DH), commonly a carnivore or omnivore, sexual development takes place. This results in the shedding of sporocysts into the environment with faecal matter. The IH gets infected by ingesting sporocysts present in contaminated water and/or food [2].

The role of DH in the epidemiology of *Sarcocystis* is of crucial significance, as they are responsible for the distribution of the infective stages into the environment [1]. This serves as a potential route of IH infection. Pathogenic *Sarcocystis* species have been reported in IHs such as wildlife, farm animals, and humans [1,3]. Because many IH species serve as important food-producing animals, this carries substantial economic significance.

Canidae is one of the most widespread families of carnivorous mammals, with more than 35 different species of wild wolves, coyotes, jackals, and foxes [4]. Red fox (*Vulpes vulpes*) is the most widely distributed of all wild canids, inhabiting nearly 70 million km^2^ in Eurasia, North Africa and the Arctic tundra regions [5,6,7]. Recent anti-rabies bait vaccinations have reduced the pressure of red fox hunting in Europe. This has led to an increase in red fox population and therefore intensifies the potential for the ectoparasites and endoparasites to spread [8,9,10,11,12]. It is a highly adaptable opportunistic animal, which is most active during twilight and night periods, and occasionally during the day, travelling approximately 10 km per day [13,14]. Red foxes have been observed to inhabit various environments, including urban ones such as city centres and areas in proximity to farms. As omnivores, the red fox’s diet includes fruits, berries, grasses, birds, small mammals, insects, and carrion [13,15]. In Europe, it was shown that a rather large part of the red fox diet consists of birds and ungulate carrion [15,16].

A wide range of adaptability to both the habitat and the food source, as well as the proximity to humans, companion animals and livestock, makes the red fox a notable animal in the parasitology context. To date, only four molecularly based reports on the red fox as a potential DH for *Sarcocystis* have been made. One study in Germany has identified *Sarcocystis capracanis*, *Sarcocystis capreolicanis*, *Sarcocystis gracilis*, *Sarcocystis miescheriana* and *Sarcocystis tenella* in the intestinal scrapings by sequencing the *18S* rRNA gene fragment [17]. In South Korea, *Sarcocystis pilosa* was determined by *18S* rRNA and cytochrome c oxidase I gene (*cox1*) sequences [18]. *Sarcocystis arieticanis*, *S. capracanis*, *Sarcocystis cruzi*, *S. miescheriana*, and *S*. *tenella* were identified in red fox intestine content in the Czech Republic [19]. Finally, in Lithuania, *Sarcocystis rileyi* was identified based on sequence analysis of the internal transcribed spacer (*ITS1*) region [20].

Investigation into the DH may reveal changes in parasite circulation and contribute to the detection of previously unrecognised *Sarcocystis* species. As numerous potential IHs of *Sarcocystis*, including ungulates and birds, are preyed upon or scavenged by red foxes in Lithuania, they may carry a wide variety of *Sarcocystis* species. Therefore, we conducted a comprehensive investigation involving a wide range of species-specific PCR primers to expand the current knowledge on red foxes’ involvement as DH of *Sarcocystis* parasites.

## 2. Materials and Methods

### 2.1. Sample Preparation

The research was conducted under the approval guidelines of the Ethics Committee of the State Scientific Research Institute Nature Research Centre (no. GGT-1; issued on 11 January 2024). The original Hunting Rules (Order No. 258 of 27 June 2000) issued by the Minister of Environment of the Republic of Lithuania were revised in 2010 (Order No. D1-768 of 15 September 2010), introducing provisions that permitted the hunting of the red fox throughout the entire year in Lithuania.

**Figure 1:**
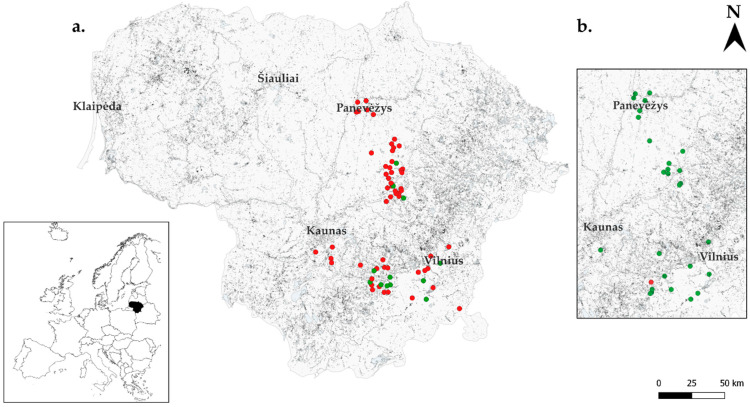
Red fox sample collection areas, showing samples testing positive (green) or negative (red) for *Sarcocystis* species determined by molecular analysis. (**a**) Samples tested for *Sarcocystis* species that use birds as IHs. (**b**) Samples tested for *Sarcocystis* species that use various ungulates as IHs.

In total, 69 red fox carcasses were collected from 2019 to 2025 on the territory of eastern Lithuania (Figure 1).

The method of collecting intestinal scrapings was adapted from the Verma et al. protocol [21]. Red foxes were dissected, and the small intestine was excised by two cuts. Extrusion expelled the faecal matter. The intestine was cut lengthwise and spread over the tray with the inner side facing up. Intestinal epithelium was lightly scraped with a scalpel, and the scrapings were diluted in 100–200 mL of distilled water. After homogenisation in a commercial blender at top speed 1–2 times, avoiding frothing, samples were put into 50 mL centrifuge tubes (nerbe plus GmbH & Co. KG, Winsen, Germany) and centrifuged using a Biosan INC-4200R refrigerated centrifuge (Biosan, Riga, Latvia) at 1600× *g* for 6 min. The supernatant was discarded; the sediment was resuspended in 100–200 mL of distilled water and homogenised again in a blender. The procedure was repeated until most of the sporocysts were released from the host tissue and the liquid became clear. The pellet was suspended in the HBSS buffer (Carl Roth GmbH & Co. KG, Karlsruhe, Germany) and filtered through two layers of gauze. The suspension was put back into the centrifuge tubes and diluted in a 5.30% sodium hypochlorite (bleach) solution (1:1 ratio), chilled on ice for 30 min. The homogenate was centrifuged, then the supernatant was discharged slowly, and additional distilled water was added. Washing of the samples was repeated until the smell of bleach was gone. The samples were stored at 4 °C until further analysis.

100 μL of resuspended sediments from each sample was analysed under 200× and 400× magnification using a Nikon Eclipse 80i microscope (Nikon Corporation, Tokyo, Japan), and the measurements of sporocysts/oocysts were recorded. To ensure reliability and reduce the likelihood of false negatives, each sample was analysed in triplicate.

### 2.2. Molecular Analysis

Total DNA was extracted from the intestinal scraping samples using GeneJET Genomic DNA Purification Kit (Thermo Fisher Scientific Baltics, Vilnius, Lithuania) according to the manufacturer’s recommendations. Nested PCR (nPCR) was performed in two steps, targeting either the *cox1* gene (for *Sarcocystis* spp. with ungulate IHs) or the *ITS1* region (for *Sarcocystis* spp. with avian IHs). The first amplification employed universal primers: SF1/SsunR3 for the *cox1* gene and SU1F/5.8SR2 for the *ITS1* region. After the initial step, second amplification followed using species-specific primers (Table 1) designed for the targeted *Sarcocystis* species.

The first group (ungulate IH) showed a high detection rate, and in total, 28 red fox samples were analysed. However, as the second group’s (avian IH) detection rate was low, to receive more precise results, an additional 41 red fox samples were tested for *Sarcocystis* spp. associated with birds.

During the first step, 25 µL of the PCR mix consisted of 12.5 µL of Taq PCR Master Mix (Vazyme Biotech Co., Nanjing, China), 0.5 µM of each forward and reverse primer, 4 µL of sample DNA (concentrations ~100 ng/µL), and 7.5 µL of nuclease-free water. While the second step of the nPCR was carried out using 12.5 µL of Taq PCR Master Mix (Vazyme Biotech Co.), 0.5 µM of each forward and reverse primer, 2 µL of the amplified nPCR product from the first step, and nuclease-free water to reach the final volume of 25 µL. For both nPCR steps, the amplification programme remained the same, consisting of an initial denaturation step at 95 °C for 5 min, followed by 35 cycles of denaturation at 95 °C for 15 s, annealing at the temperature based on the annealing temperature of the primer pair (Table 1) for 15 s and extension at 72 °C for 1 min. The final extension was carried out at 72 °C for 5 min. The success of the nPCR was tested using 1.00% TopVision Agarose (Thermo Fisher Scientific Baltics) agarose gel electrophoresis using a Biocom MP-300V electrophoresis system (Biocom Ltd, UK) and gels were visualised using a Biometra darkroom hood (Biometra GmbH, Göttingen, Germany). Positive and negative controls were included in each PCR run. Nuclease-free water, instead of a DNA template, served as a negative control. DNA samples from several *Sarcocystis* species, stored in the collection of the Laboratory of Molecular Ecology at the State Scientific Research Institute Nature Research Centre and previously confirmed by Sanger sequencing, were used as positive controls.

Each positive sample was cleaned before sequencing. The mixture consisted of 0.5 µL of exonuclease I (Exo I, Thermo Fisher Scientific Baltics), 1 µL of alkaline phosphatase FastAP (Thermo Fisher Scientific Baltics) and 5 µL of PCR product. The bidirectional Sanger sequencing was performed using a Big-Dye^®^Terminator v3.1 Cycle Sequencing Kit (Thermo Fisher Scientific Baltics) and a 3500 Genetic Analyzer (Applied Biosystems, Foster City, CA, USA).

### 2.3. Data Analysis

Obtained sequences were compared to the most closely related *Sarcocystis* spp. in the NCBI GenBank database using the Nucleotide BLAST tool [28] (http://blast.ncbi.nlm.nih.gov/, accessed on 15 November 2025). A phylogenetic relationship tree was created using MEGA12 (version 12.0.14) software. The evolutionary history was inferred by using the Maximum Likelihood method based on the Tamura–Nei model and 1000 bootstrap replications [29]. The significance of the difference in detection rates between microscopical and molecular results of 28 samples was assessed using a Z-pooled unconditional exact test [30].

Sterne’s method (Quantitative Parasitology v 3.0 software [31]) was used with 95.00% confidence intervals (CI) for the frequency of listed *Sarcocystis* spp. in DH animals. The Shannon diversity index *H* and Pielou’s evenness index *J* were calculated (Excel, Microsoft Corporation, Redmond, WA, USA, 2021) and used to analyse *Sarcocystis* species prevalence in DH. Shannon *H* is not limited but depends on the number of species, and the more species there are the higher it is. Pielou’s *J* is species evenness, with 0 being the most uneven populations (dominance of one species), and 1 being the most even distribution of the species. To simplify the comparison, we divided *Sarcocystis* species into two groups by their IH: “Wild”, which includes mainly *Sarcocystis* species that use free-ranging Cervids as IH and “Domestic”, with farmed ungulates as IH. It should be noted that goats and horses in Lithuania are strictly domestic animals; therefore, *S. bertrami* and *S. capracanis* are included in the “Domestic” group. *Sarcocystis miescheriana* and *S. cruzi* were excluded from the analysis as they cannot be categorised into these groups, since they can infect both domestic and wild hosts. To provide a basis for comparison raccoon dog (*Nyctereutes procyonoides*) [32] and grey wolf (*Canis lupus*) [33] data from our previous studies were included in the analysis as these samples were collected from geographically proximate locations and tested for the same *Sarcocystis* species.

## 3. Results

### 3.1. Light Microscopy

**Figure 2:**
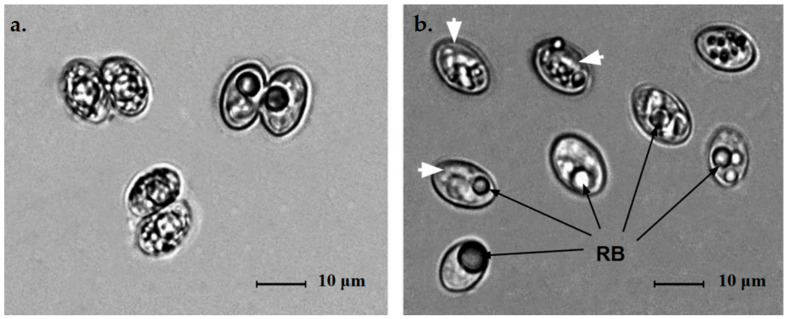
Appearance of oocysts/sporocysts of *Sarcocystis* spp. in the epithelium of the small intestines of the red fox from Lithuania. (**a**) Sporulated oocysts; (**b**) sporocysts. Sporocysts with well-defined sporozoites (white arrow) and a compact residual body (RB).

Microscopic examination of red fox intestinal samples revealed the presence of sporulated oocysts and/or free sporocysts under a light microscope (LM) (Figure 2).

Ellipsoidal sporulated oocysts (Figure 2a) were thin-walled, contained two sporocysts, and measured 18.59–26.99 × 8.12–14.74 μm (19.43 ± 2.62 μm × 13.01 ± 2.07; *n* = 130). Free sporocysts (Figure 2b) were 12.82–18.84 × 7.52–10.07 μm (13.44 ± 1.26 × 9.5 ± 0.89 μm; *n* = 200) in size.

The number of sporulated oocysts and/or free sporocysts detected in the 24 × 24 mm coverslip ranged from 1 to 320. Free sporocysts were detected in 21 out of 28 initial intestinal samples (75.00%; CI 55.45–88.05%) that were tested for both groups of *Sarcocystis* species. An unconditional exact test indicated that molecular analysis achieved a significantly higher detection rate of *Sarcocystis* spp. than microscopic analysis (*p* = 0.0259). During the molecular analysis, an additional 41 samples were included to increase the sample size for testing *Sarcocystis* that use birds as IHs, and these samples were also examined microscopically. In a total of 69 red foxes examined, oocysts/sporocysts of *Sarcocystis* spp. were observed in 47 (68.12%) of the samples.

### 3.2. Molecular Identification

**Figure 3:**
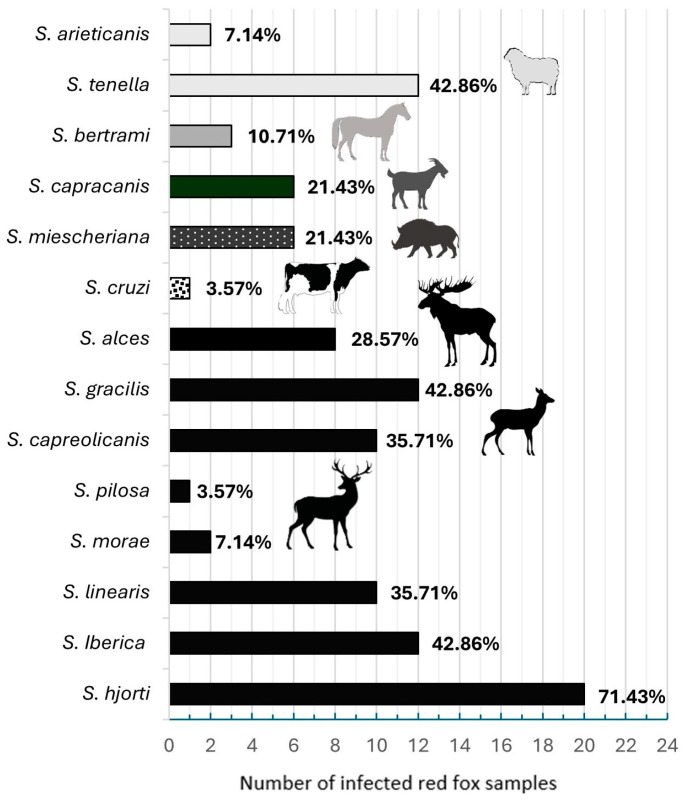
Infection rates of *Sarcocystis* spp. in intestinal samples of red foxes. Symbols (animals) indicate the IH of each *Sarcocystis* species, with black shading grouping species that utilise Cervidae as IHs.

Out of 28 initially examined red fox samples, 27 (96.43%; CI 82.52–99.81%) were found to be infected with at least one *Sarcocystis* species that uses ungulates as IHs. In total, 14 different *Sarcocystis* species were identified: *Sarcocystis alces*, *S. arieticanis*, *Sarcocystis bertrami*, *S. capracanis*, *S. capreolicanis*, *S. cruzi*, *S. gracilis*, *Sarcocystis hjorti*, *Sarcocystis iberica*, *Sarcocystis linearis*, *S. miescheriana*, *Sarcocystis morae*, *S. pilosa,* and *S. tenella*. The most frequently detected species was *S. hjorti,* identified in 20 of 28 samples (71.43%). *Sarcocystis gracilis*, *S. iberica* and *S. tenella* were also commonly observed, each present in 12 of 28 individuals. The least common species, *S. cruzi* and *S. pilosa*, were detected only once (Figure 3). Two tested species that utilise Cervidae as IHs, *Sarcocystis venatoria* and *Sarcocystis taeniata*, were not detected in any of the samples.

In contrast, screening of the initial 28 red fox samples using nPCR targeting *ITS1* for *Sarcocystis* species with avian IHs revealed a relatively low infection rate, with positive detections in five out of 28 samples. After testing an additional 41 red foxes, 16/69 samples (23.19%) were found to be infected. Two previously known *Sarcocystis* species were observed in the investigated samples. *Sarcocystis rileyi* was found in four individuals, while *Sarcocystis albifronsi* was found only in two specimens. In addition, two potentially genetically distinct yet uncharacterised *Sarcocystis* species were detected and, in this study, were temporarily named *Sarcocystis* sp. 1 (PX619817–PX619818) and *Sarcocystis* sp. 2 (PX619819–PX619829). In total, two isolates of *Sarcocystis* sp. 1 were found, whose *ITS1* sequences had no analogues in the NCBI GenBank database and were most closely related to *Sarcocystis wenzeli* and *Sarcocystis cristata* (91.62% and 90.54%, respectively). Another 11 isolates were assigned as *Sarcocystis* sp. 2 (796 bp), which had 100% nucleotide sequence similarity to previously found isolates from the intestines of European pine marten (PV364797.1; 461 bp) [24]. Compared to each other, *Sarcocystis*. sp. 1 and *Sarcocystis*. sp. 2 had 93.58% nucleotide sequence similarity of the *ITS1* region.

All positive samples were identified by sequencing amplified partial regions of the *cox1* gene of *Sarcocystis* species that use ungulates as IH or *ITS1* region fragments for the species that use avian as IH. These sequences were deposited in GenBank under accession numbers PX548616–PX548720 for *Sarcocystis* spp. associated with ungulate IHs and PX619811–PX619829 for *Sarcocystis* spp. associated with avian IHs. Comparisons of these sequences, as well as with the sequences of the most closely related species, are shown in Table 2.

**Figure 4:**
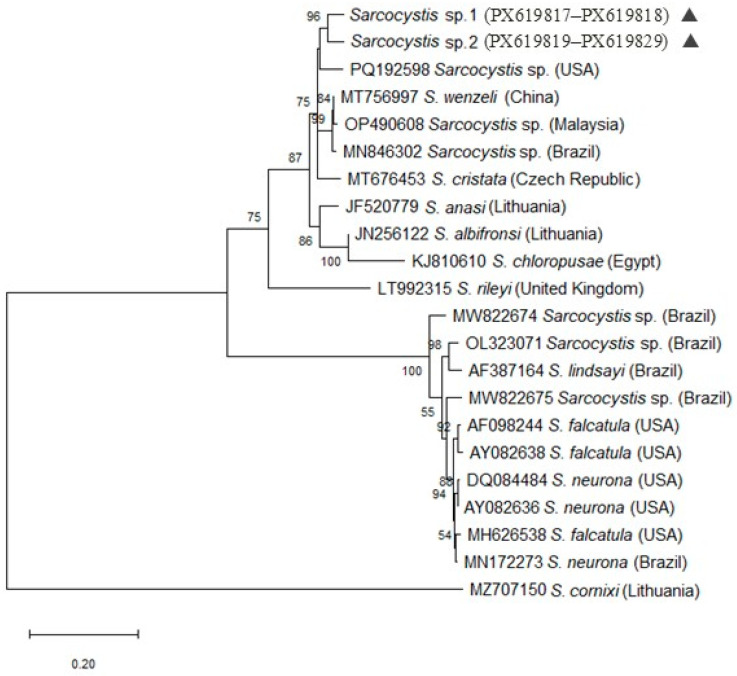
Phylogenetic tree of selected *Sarcocystis* species based on *ITS1* nucleotide sequences. Sequences obtained during this study are highlighted using triangles. The Maximum Likelihood method based on the Tamura–Nei model and 1000 bootstrap replications was adopted to infer the evolutionary history. The numbers on the branch represent the value of the strength of statistical significance; values below 50 are hidden. The tree was rooted using a distantly related species that uses birds as IH.

A phylogenetic tree was constructed using *ITS1* sequences from GenBank to identify the interspecific relationships for our *Sarcocystis* sp. 1 and *Sarcocystis* sp. 2 (Figure 4). Our isolates formed a cluster which included a group with unidentified *Sarcocystis* species from *Canis lupus* in the USA, Brazil, Malaysia, and *S*. *wenzeli* from China and *S*. *cristata* from the Czech Republic. Notably, a high bootstrap value of 96 was observed in the cluster with two *Sarcocystis* species detected and described in the current study.

**Figure 5:**
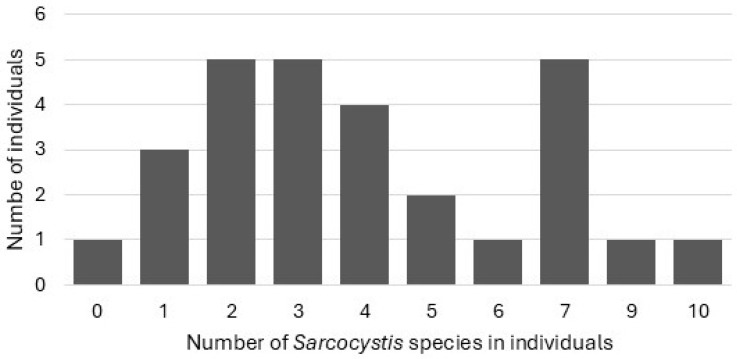
Coinfection level of *Sarcocystis* species in red fox samples that use ungulates as IH.

Among samples positive for *Sarcocystis* species linked to ungulate IHs, three individuals had a single species infection. *Sarcocystis* species coinfections were observed in 85.71% (24/28) of samples. Notably one animal harboured 10 *Sarcocystis* species: *S. alces*, *S. bertrami*, *S. capracanis*, *S. capreolicanis*, *S. hjorti*, *S*. *iberica*, *S. linearis*, *S. miescheriana*, *S. morae*, and *S*. *tenella* (Figure 5).

Whereas *Sarcocystis* spp. with avian IH coinfections were rarely detected in the analysed samples, only sixteen (23.19%) tested positive. Only three samples (4.35%) were infected with two *Sarcocystis* species, and the remaining 13 (18.84%) were infected with a single species.

Further distribution analysis of *Sarcocystis* species that use ungulates as IH was performed using the Shannon diversity index (H) and Pielou’s evenness index (*J*). According to Pielou’s evenness (*J* = 0.90 in “Wild” and 0.93 in “Domestic”) the *Sarcocystis* species were evenly distributed. However, Shannon’s diversity index (*H* 1.87 in “Wild” and 1.29 in “Domestic”) indicates higher species diversity of *Sarcocystis* species in the “Wild” group.

## 4. Discussion

Molecular detection yielded a higher overall detection rate (96.43%) compared to light microscopy (75.00%); however, it should be noted that molecular analysis targeted only *Sarcocystis* species associated with ungulates, whereas LM covered all detected *Sarcocystis* species. Due to uneven distribution between the *Sarcocystis* species with avian IH and LM results, the smaller number of *Sarcocystis* spp. is represented. Intraspecific and interspecific nucleotide sequence similarities did not overlap, showing that the chosen genetic markers are suitable for species differentiation. Although a species-specific PCR primer approach is expensive, it is unavoidable given how common mixed *Sarcocystis* infections are in their DH [32,33,34].

There is limited information on the identification of *Sarcocystis* spp. using intestinal scrapings from red foxes, in Europe as well as worldwide. In the study from the Czech Republic, out of 50 red fox samples analysed molecularly, 21 (42.00%) samples were found to be positive for at least one of five *Sarcocystis* species (*S. arieticanis* (2.00%), *S. capracanis* (2.00%), *S. cruzi* (12.00%), *S. miescheriana* (12.00%), and *S*. *tenella* (24.00%)) [19]. In a study from South Korea, a single specimen of red fox was analysed. Gene amplification targeted *18S* rRNA and *cox1* genes, and *S. pilosa* was found [18]. In a study from Germany, 50 samples were analysed, and molecular examination revealed a prevalence of 38.00%. The following *Sarcocystis* species were identified: *S. tenella* or *S. capracanis* (10.00%), *S. miescheriana* and *S. gracilis* (8.00% of samples each), *S. albifronsi* or *Sarcocystis anasi* (6.00% each) and *S. capreolicanis* (4.00%) [17]. In the study from Lithuania, 23 faecal and 20 intestinal samples were analysed for the presence of *S. rileyi*, and three intestinal samples (15.00%) were found to be positive [20].

Notably, only one of the studies used the same methodology for sample preparation [20]. Intestinal scrapings allow thorough investigation, as a larger area can be analysed compared to faecal examination. Therefore, it may justify the much higher (96.00%) *Sarcocystis* detection rate in our study. We were also targeting the *cox1* and *ITS1* regions, as the conservative nature of ribosomal genes prevents distinction of more closely related species [35,36], which likely increased detection rates and identification of certain *Sarcocystis* species. This is the first report of the presence of seven *Sarcocystis* species in intestinal red fox samples: *S. alces*, *S. bertrami*, *S. capreolicanis*, *S. hjorti*, *S*. *iberica*, *S. linearis*, and *S. morae*. In addition, nine *Sarcocystis* species previously reported in other studies were also detected in the present study: *S. arieticanis*, *S. capracanis*, *S. cruzi*, *S. gracilis*, *S. miescheriana*, *S*. *tenella*, *S. pilosa*, *S. albifronsi* and *S. rileyi*. Surprisingly, neither *S. venatoria* nor *S. taeniata* were found in the analysed 28 red fox samples, despite both species having been reported in grey wolves from Lithuania [33]. *Sarcocystis taeniata* has also been identified in the raccoon dog samples from the same region [32].

There is evidence that red foxes and mustelids may coexist within the same ecosystems and therefore potentially compete for food resources [37,38]. In Lithuanian studies, several *Sarcocystis* species previously reported in different mustelids were also detected in the intestinal samples from red foxes in our study, including *S. arieticanis*, *S. bertrami*, *S. capracanis*, *S. capreolicanis*, *S. cruzi*, *S. hjorti*, *S. morae*, *S. linearis*, and *S. rileyi* [24,27,39,40].

In contrast to the high infection rate of *Sarcocystis* species with ungulate IHs, those with avian IHs were rarely detected in red fox samples. Although red foxes are omnivorous and are known to consume ducks, *S. anasi* and *S*. *wenzeli* were not detected in any of the 69 samples analysed.

In *Sarcocystis* research, observed interspecific differences of 8% or more indicate that the analysed sequences belong to different species (Table 2). Intraspecific comparison could not be made because *Sarcocystis* sp. 1 has no closely related sequences in GenBank. Comparison of *Sarcocystis* sp. 1 with the closest species revealed an 8.39% sequence divergence providing a basis for designation as a separate species. *ITS1* region is highly variable; further studies incorporating additional genetic markers are needed. Although we have found that red fox serves as a DH for *Sarcocystis* sp. 1 and *Sarcocystis* sp. 2, for further description and formal taxonomic recognition of the species, it is crucial to investigate sarcocysts from IH tissues. The current evidence suggests that these sequences may represent a distinct, previously uncharacterised species. *Sarcocystis* sp. 2 had 100% similarity with a shorter *ITS1* gene fragment of previously reported isolates (QC 58%) from the intestines of European pine marten [24].

When comparing *Sarcocystis* sp. 1 and *Sarcocystis* sp. 2 sequences their similarity (93.58%) is distant enough for them to be considered as separate species. Phylogenetic analysis revealed a strong relationship between *Sarcocystis* sp. 1 and *Sarcocystis* sp. 2. Nevertheless, there is still a need for further investigation, as additional data on isolates provide greater insights into the phylogenetic relationships of these species.

We used the data from two of our previous studies to compare the red fox to the other two canids (Table 3) [32,33]. Relatively high evenness of *Sarcocystis* species was observed across all host species (*J* = 0.90–0.99), indicating a balanced distribution of parasite species within samples. All three predatory mammals showed higher diversity of *Sarcocystis* species associated with wild IHs compared to those associated with domestic IHs. The highest overall *Sarcocystis* diversity was estimated in grey wolves, followed by red foxes and raccoon dogs. Moreover, raccoon dogs were distinguished by reduced diversity of *Sarcocystis* species associated with domestic IHs.

Moderately high levels of diversity indices of *Sarcocystis* species in red foxes might indicate multiple types of feeding habitats. *Sarcocystis* species usually demonstrate host specificity within IH, generally infecting a limited range of specific animal species. Red foxes are the most widespread member of Canidae and inhabit a variety of areas, including both deserted tundra, forests, meadows, and populated city centres [5,6,7]. In raccoon dogs, the lower index depicts a less variable community of *Sarcocystis* species that use ungulates as IH. This might be the result of their habitat choice, which is reflected by the availability of good coverage areas. While red foxes may prefer open spaces such as farm fields, raccoon dogs tend to avoid areas without sufficient cover [41]. As both raccoon dogs and red foxes are opportunistic carnivores and mesopredator, their diet may overlap, although the competition is not high [16]. The grey wolf had a high (*H* > 2) diversity level in the “Wild” and moderate in the “Domestic” group species, and the highest coefficient between these canids overall (see Table 3 ). The grey wolf is an apex predator and often hunts larger prey. *Sarcocystis* species infection level and diversity in grey wolves indicate a higher exposure rate. The red fox has previously been observed to be attracted towards grey wolf-killed ungulate carcasses, indicating facilitation rather than competition-driven avoidance of this apex predator [42].

In addition, we have calculated Pielou’s evenness index (*J*) for all the groups (“Wild” and “Domestic” IH) separately, and all of them show numbers between 0.90 and 0.99. We may conclude that none of the *Sarcocystis* species found was dominant in our three examined canid species. It might be an indication that none of the *Sarcocystis* species has high host specificity to these canids as DH.

The high detection rate of *Sarcocystis* in red foxes is unsurprising, given that red fox populations are abundant in Lithuania. As an opportunistic omnivore, the red fox can adapt its dietary preferences based on the availability of food sources. Consumption of a wide range of different animals that are IHs leads to exposure to a wide variety of *Sarcocystis* species. Red foxes are active during the twilight and night periods, and on occasion during the day, travelling approximately 10 km per day [13,14], allowing them to range across both natural and agricultural landscapes. Given that red foxes are DH of *Sarcocystis* species, larger fox populations may elevate the risk of transmission to livestock. This, in addition to a wide range of found species and a very high rate of mixed coinfections, shows that the red fox has an important role in contributing to the *Sarcocystis* life cycle. It also highlights the fox’s contribution to facilitating the spread of these parasites in the environment. It is important to conduct further investigation into *Sarcocystis* species that utilise red foxes to broaden the data on currently known and possibly novel *Sarcocystis* spp.

## 5. Conclusions

The present study demonstrates that red foxes in Lithuania are exposed to a highly diverse range of *Sarcocystis* species, with parasites utilising ungulates as IHs being considerably more common than those associated with avian IHs. This finding represents potential dietary preferences of red foxes in Lithuania.

Based on nested PCR and Sanger sequencing, seven previously described *Sarcocystis* species (*S*. *alces*, *S*. *bertrami*, *S*. *capreolicanis*, *S*. *hjorti*, *S*. *iberica*, *S*. *linearis*, and *S*. *morae*) were identified in red foxes for the first time. In addition, seven *Sarcocystis* species with ungulate IH (*S. arieticanis*, *S. capracanis*, *S. cruzi*, *S. gracilis*, *S. miescheriana*, *S*. *tenella* and *S. pilosa*) and two with avian IH (*S. albifronsi* and *S. rileyi*), which were previously reported in other studies, were also detected in the present study. In addition, two potentially novel *Sarcocystis* taxa (tentatively named *Sarcocystis* sp. 1 and *Sarcocystis* sp. 2) were detected; however, further morphological and molecular characterisation incorporating additional genetic markers is required for species delimitation.

Despite the relatively small sample size, our results show high prevalence and diversity of *Sarcocystis* spp. in red foxes. These preliminary results indicate that they are important DHs contributing to the maintenance and circulation of these parasites in natural ecosystems. The broad panel of species-specific primers used in the present study enabled a comprehensive assessment of *Sarcocystis* species richness in red foxes and provided a valuable framework for comparative studies of *Sarcocystis* distribution in various predatory mammals from different geographical regions.

## Figures and Tables

**Table 1:** Primer pairs used for molecular analysis.

Primer	Sequence	Step	Region	Length bp	T_a_	Target Species	DH/IH
SF1 ^1^	ATGGCGTACAACAATCATAAAGAA	I	*cox1*	919	51	*Sarcocystis* spp.	Canid/ Ungulate
SsunR3 ^2^	CCGTTGGWATGGCRATCAT						
V2alc3 ^3^	CCTAGGTACCGTGCTCTTTGATG	II	*cox1*	398	63	*S. alces*	Canid/ Cervid
V2alc4 ^3^	CTTCGAGGCCAGTAGTTACCATA						
Arie7F ^2^	TAATTTCCTGGGTACTGTACTGTTTG	II	*cox1*	290	64	*S. arieticanis*	Canid/ Sheep
Arie7R ^2^	TACTTACGCATTGCGATATTACG						
V2ber7 ^4^	CCCCACTCAGTACGAACTCC	II	*cox1*	381	59	*S. bertrami*	Canid/ Horse
V2ber8 ^4^	ACTGCGATATAACTCCAAAACCA						
V2ca3 ^5^	ATACCGATCTTTACGGGAGCAGTA	II	*cox1*	501	63	*S. capracanis*	Canid/ Goat
V2ca4 ^5^	GGTCACCGCAGAGAAGTACGAT						
V2capreo1 ^3^	CATCGTAGAGCCCCGTACTC	II	*cox1*	416	59	*S. capreolicanis*	Canid/ Cervid
V2capreo2 ^3^	ACCGCTATACGCTGGAGCTG						
V2cr7 ^3^	CAATGTGCTGTTTACGCTCCA	II	*cox1*	501	57	*S. cruzi*	Canid/ Cattle
V2cr8 ^3^	TCGTACAGGCCCGTAGTTAG						
V2gr9 ^3^	GTGCTCGGGGCAGTGAAC	II	*cox1*	410	55	*S. gracilis*	Canid/ Cervid
V2gr10 ^3^	GCCAGTAGTCATCATGTGGTGT						
V2hjo1 ^3^	AAGGTACACGGCATTGTTCAC	II	*cox1*	268	57	*S. hjorti*	Canid/ Cervid
V2hjo2 ^3^	GAAAACTACCCTGCCGCCTA						
V2ibeven1 ^3^	ATGGGCCATTATATTTACTGCTCTG	II	*cox1*	252	59	*S. iberica* *S. venatoria*	Canid/ Cervid
V2ibeven2 ^3^	GCCGCCAAAAACTACCTTACC						
V2ven3 ^3^	TGCTCAGGCATTGGGATATAA	II	*cox1*	308	55	*S. venatoria*	Canid/ Cervid
V2ven4 ^3^	CGTAGACTGCATGACGACTTACAA						
V2mie5 ^4^	TCCTCGGTATTAGCAGCGTACTG	II	*cox1*	358	55	*S. miescheriana*	Canid/Swine
V2mie6 ^4^	ATTGAAGGGCCACCAAACAC						
V2mor1 ^5^	GTGTGCTTGGATCGGTCAAC	II	*cox1*	332	57	*S. morae*	Canid/ Cervid
V2mor2 ^5^	GCCGAATACCGGCTTACTTC						
V2pil3 ^3^	GTTAATTTCCTGGGCACAGTGTT	II	*cox1*	291	59	*S. pilosa*	Canid/ Cervid
V2pil4 ^3^	CGAAAACTACTCTGCCGCCTAC						
V2taelin1 ^3^	CGTAGACTGCATGACGACTTACAA	II	*cox1*	662	55	*S. taeniata*, *S. linearis*	Canid/ Cervid
V2taelin2 ^3^	CAAAGATGGATTTGCTGCCTA						
Ten8F ^5^	ATACCGCTCTACGCTGGATCTAC	II	*cox1*	421	66	*S*. *tenella*	Canid/ Sheep
Ten8R ^5^	AACCATCGTACAATCCAAAACTAAA						
Su1F ^1^	GATTGAGTGTTCCGGTGAATTATT	I	*ITS1*	700–1200	59	*Sarcocystis* spp.	Carnivora/ Aves
5.85R2 ^1^	AAGGTGCCATTTGCGTTCAGAA						
GsSrilF2 ^6^	ACGTTGTTCTATATTATGTGACCATT	II	*ITS1*	663	60	*S. rileyi*	Carnivora/ Aves
GsSrilR2 ^6^	TACTATAGAGGTGAAAGGGAGGTGA						
AZVF1 ^6^	TCAAAACGTCCAAATAATGGTAT	II	*ITS1*	830–850	55	*S. albifronsi*, *S. anasi*, *S*. *wenzeli*	Carnivora/Aves
AZVR1 ^6^	ACACATTCCTACTGCCTTCCAC						
GsSanaF3 ^7^	ACAAGTGTGCGTTCCTGTCG	II	*ITS1*	559	57	*S. anasi*	Canid/Anseriformes
GsSana R3 ^7^	TGTGTAGAAAACAGATGGAACG						
GsSalb F3 ^7^	GCACAACTGTGTATGCCTGTCT	II	*ITS1*	435	55	*S. albifronsi*	Canid/Anatidae
GsSalb R3 ^7^	ATTCTTGTCACCACCTCACC						

^1^ Gjerde et al., 2013 [22], ^2^ Marandykina-Prakienė et al., 2022 [23], ^3^ Prakas et al., 2025 [24], ^4^ Baranauskaitė et al., 2022 [25], ^5^ Marandykina-Prakienė et al., 2023 [26], ^6^ Prakas et al., 2023 [27], ^7^ primer pairs designed specifically for this study.

**Table 2:** Sequence similarity analysis of *Sarcocystis* species.

*Sarcocystis* Species	Gene Bank acc. n.	Number of Genotypes	Similarity with Same Species, %	Query Coverage, %	Similarity with Closely Related Species, %	Query Coverage, %
*S. alces*	PX548616–PX548623	3	97.16–100	100	*S. capracanis* (83.33–85.29)	95–97
*S. arieticanis*	PX548624–PX548625	2	90.87–99.59	100	*S*. *rossii* (81.25–81.73)	86
*S. bertrami*	PX548626–PX548628	2	96.75–100	99–100	*S*. *suihominis* (77.38–78.87)	97
*S. capracanis*	PX548629–PX548634	6	96.48–100	100	*S*. *tenella* (91.10–93.97)	99
*S. capreolicanis*	PX548635–PX548644	1	97.87–101	99–100	*S. alceslatrans* (94.95–95.48)	100
*S. cruzi*	PX548645	1	98.91–100	100	*S*. *levinei* (92.51–93.25)	99–100
*S. gracilis*	PX548646–PX548657	2	98.65–100	100	*S. capracanis* (82.75–84.64)	94–100
*S. hjorti*	PX548658–PX548677	2	99.12–100	100	*S. pilosa* (92.48–94.25)	100
*S*. *iberica*	PX548678–PX548689	2	99.03–100	100	*S. venatoria* (88.41–97.58)	100
*S. linearis*	PX548690–PX548699	6	98.38–100	100	*S. taeniata* (96.60–97.73)	100
*S. miescheriana*	PX548700–PX548705	2	98.73–100	100	*S*. *sigmoideus* (76.99–77.78)	70–75
*S. morae*	PX548706–PX548707	2	96.23–100	100	*S*. *cervicanis* (83.90–85.27)	100
*S. pilosa*	PX548708	1	97.05–100	100	*S. hjorti* (91.14–93.36)	100
*S*. *tenella*	PX548709–PX548720	5	97.86–100	100	*S. capracanis* (89.54–94.37)	94–100
*Sarcocystis* sp. 1	PX619817–PX619818	1	–	–	*S*. *wenzeli* (90.31–91.61)	99
*Sarcocystis* sp. 2	PX619819–PX619829	1	100	58	*S*. *wenzeli* (90.67–91.16)	100
*S. albifronsi*	PX619811–PX619812	1	100	100	*S. anasi* (91.27)	91
*S. rileyi*	PX619813–PX619816	1	99.51	100	*S*. *atraii* (91.58–92.11)	100

**Table 3:** Comparison of diversity and evenness of *Sarcocystis* spp. associated with ungulates detected in the intestines of red foxes, raccoon dogs, and grey wolves from Lithuania.

	Red Fox (*Vulpes vulpes*)		Raccoon Dog (*Nyctereutes procyonoides*)		Grey Wolf (*Canis lupus*)	
	“Wild”	“Domestic”	“Wild”	“Domestic”	“Wild”	“Domestic”
Pielou’s evenness *J*	0.90	0.93	0.93	0.99	0.97	0.94
Shanon’s diversity *H*	1.87	1.29	1.93	0.69	2.04	1.54

## Data Availability

The *cox1* gene sequences of *Sarcocystis* spp. have been deposited in GenBank under accession numbers PX548616–PX548720, while *ITS1* sequences of *Sarcocystis* spp. are available under accession numbers PX619811–PX619829.

## Abbreviations

The following abbreviations are used in this manuscript:

IH	Intermediate host
DH	Definitive host
LM	Light microscopy
*cox1*	Cytochrome c oxidase I
*ITS1*	Internal transcribed spacer
